# Outcome contingency selectively affects the neural coding of outcomes but not of tasks

**DOI:** 10.1038/s41598-019-55887-0

**Published:** 2019-12-18

**Authors:** David Wisniewski, Birte Forstmann, Marcel Brass

**Affiliations:** 1 Department of Experimental Psychology, Ghent University, Ghent, Belgium; 2 Integrative Model-Based Cognitive Neuroscience Research Unit, University of Amsterdam, Amsterdam, The Netherlands

**Keywords:** Cognitive control, Decision, Motivation, Human behaviour

## Abstract

Value-based decision-making is ubiquitous in every-day life, and critically depends on the contingency between choices and their outcomes. Only if outcomes are contingent on our choices can we make meaningful value-based decisions. Here, we investigate the effect of outcome contingency on the neural coding of rewards and tasks. Participants performed a reversal-learning paradigm in which reward outcomes were contingent on trial-by-trial choices, and performed a ‘free choice’ paradigm in which rewards were random and not contingent on choices. We hypothesized that contingent outcomes enhance the neural coding of rewards and tasks, which was tested using multivariate pattern analysis of fMRI data. Reward outcomes were encoded in a large network including the striatum, dmPFC and parietal cortex, and these representations were indeed amplified for contingent rewards. Tasks were encoded in the dmPFC at the time of decision-making, and in parietal cortex in a subsequent maintenance phase. We found no evidence for contingency-dependent modulations of task signals, demonstrating highly similar coding across contingency conditions. Our findings suggest selective effects of contingency on reward coding only, and further highlight the role of dmPFC and parietal cortex in value-based decision-making, as these were the only regions strongly involved in both reward and task coding.

## Introduction

Making decisions is an integral part of our life. Most of these choices are value-based, i.e. they are made with expected outcomes in mind. Value-based choices are made in separate stages: we first evaluate all options, and then select the option with the highest subjective value^1^. After implementing the chosen behavior^2^, predicted and experienced outcomes are compared, and reward prediction errors are computed^3–5^. This dopamine-mediated learning signal^6^ indicates the need to update our internal models of action-outcome contingencies, which then leads to an adaption of future behavior.

This process is modulated by various properties of choice outcomes, e.g. their magnitude^7^. However, one crucial aspect has received little attention in the past: to which degree our choices directly control possible outcomes. Clearly, whether or not we believe our choices to directly *cause* their outcomes affects decision-making considerably. If we know that a specific behavior predictably leads to a desired outcome, we will choose it more often^8^. If we know that our behavior and desired outcomes are only weakly correlated, or not correlated at all, we might not prioritize any specific behavior. Here, we focus on investigating the effects of high vs low control of the outcomes of one’s own choices.

In principle, varying degrees of control of choice outcomes can affect two key processes: outcome valuation and the implementation of chosen behavior. Some previous research in non-human primates^9^, and humans^10,11^ demonstrated that choice-contingent outcomes are processed differently than non-contingent outcomes. Importantly, one might expect similar effects on neural representations of the chosen behavior that is operational for receiving the reward as well. In general, when we form an intention to perform a specific task, we need to focus on that intention and not get distracted by task-irrelevant information^12^. The need for such “goal shielding” can be expected to be stronger if outcomes are contingent on performing a specific action. In this case, mistakenly performing the wrong action is potentially costly. If outcomes are not contingent on specific actions, the need for shielding is lower as e.g. performing the wrong action has no effects on received outcomes (see^13^ for a related argument, but^14^). Previous work demonstrated that implementation of chosen actions is supported by a brain network including the frontopolar^15^, lateral prefrontal and parietal cortex^16–18^. Some initial evidence suggests that rewarding correct performance indeed enhances neural task representations^19^, but this work did not address the issue of varying degrees of control over choice outcomes.

Here, we report an experiment investigating the effects of control over choice outcomes on value-based decision making. We used a value-based decision paradigm and multivariate pattern analysis methods (MVPA^20^) to assess the effects of reward contingency (choice-contingent vs. non-contingent rewards) on valuation and, more importantly, on choice implementation. We first hypothesized that reward contingency affects the neural coding of outcomes^9,11^. We further assessed whether implementation of chosen behavior (i.e. coding of chosen tasks) is similarly affected by contingency. We hypothesized that the lateral prefrontal cortex, and especially the parietal cortex play a key role in the implementation of chosen behavior. The parietal cortex represents chosen tasks and actions^1,17^, subjective stimulus and action values^21,22^, as well as associations between choice options and their outcomes^23^. Here, we tested whether task representations in these brain regions were enhanced when rewards were choice-contingent vs when they were not.

## Results

**Figure 1:**
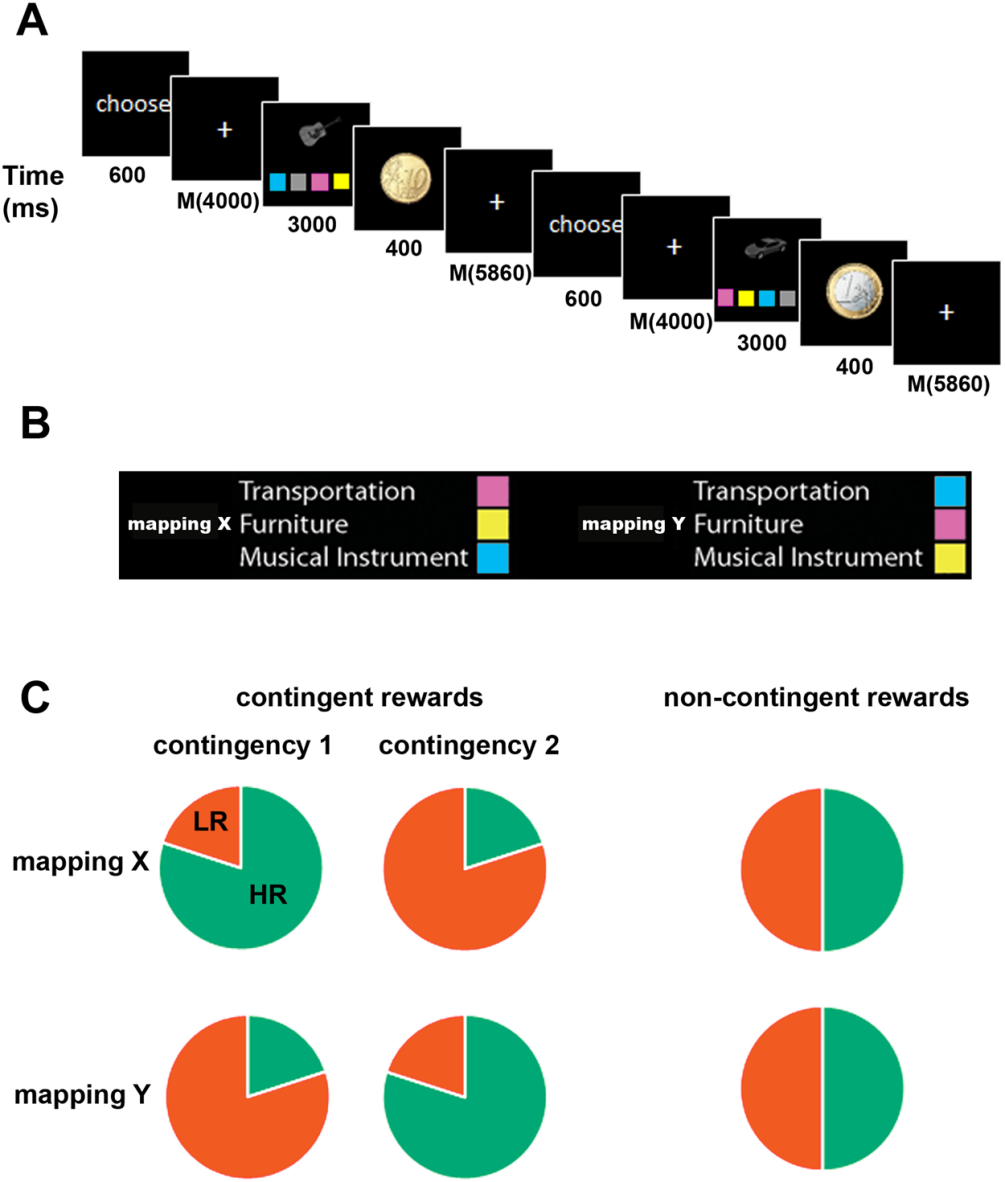
Experimental paradigm. (**A**) Trial structure. Each trial started with the cue ‘choose’ presented on screen, indicating that subjects should now decide which of the two SR mappings (mapping X or mapping Y) to perform in that trial. After a variable delay, the task screen was presented for a fixed duration, and participants implemented the chosen task. Reward feedback was presented subsequently after each trial (high reward = 1€, low reward = 10€cents, no reward). All trials were separated by variable inter-trial-intervals. (**B**) Tasks. Subjects were instructed to identify the category of a visual object presented on screen (means of transportation, furniture, musical instruments). Each category was associated with a colored button, and subjects were instructed to press the corresponding button. Two different sets of stimulus-response mappings were learned, and labelled mapping X and mapping Y. On each trial, subjects had the free choice which of the two mappings to implement. (**C**) Reward conditions. In contingent trials, subjects performed a probabilistic reversal-learning paradigm. In each trial, one of the two mappings yielded a high reward with a high probability (80%), and a low reward with a low probability (20%). The other task had the opposite reward contingencies. Which task yielded higher rewards depended on the current reward contingency, which changed across the experiment. In non-contingent trials, subjects also received high and low reward outcomes, which were assigned randomly (50%/50%) and were not contingent on specific task choices.

In every trial of this experiment, participants freely chose between performing one of two tasks (different stimulus-response (SR) mappings labelled mapping X and mapping Y, Fig. 1), and received either a high or a low reward for successful performance. Each trial started with a ‘choose’ cue being presented on screen, indicating that subjects should now choose which of the two mappings/tasks they want to perform in the current trial. After a variable delay phase, they were presented with a task screen and implemented the chosen task. Then, reward-feedback was presented. In each run of this experiment, participants performed two blocks of a probabilistic reward reversal-learning paradigm (contingent reward (CR) trials, see^24^). They chose either mapping X or mapping Y in each trial, and received reward feedback after successful performance. Crucially, reward outcomes were contingent on the specific choice made in the trial, and contingencies changed across the course of the experiment. Each run also contained two blocks of a ‘free choice’ paradigm (non-contingent reward (NCR) trials). Participants again chose between mapping X or mapping Y, and received reward feedback in each trial. Importantly, outcomes were not contingent on task choices in NCR trials, giving participants no means of controlling the reward outcomes in these trials. Experimental design was optimized to perform MVPA analyses of task coding, and reward signals were analyzed using the same method to better compare results.

### Behavioral results

**Figure 2:**
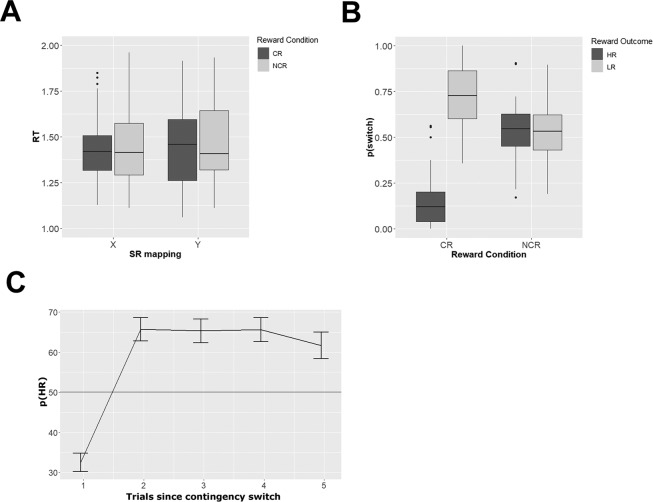
Behavioral Results. (**A**) Reaction Times (RT). The box plots depict reaction times for each combination of stimulus-response mapping and reward condition. Contingent (CR) trials are shown in dark grey, non-contingent (NCR) trials are shown in light grey. (**B)** Switch probabilities. Probability to switch away from the current task as a function of previous reward (high = HR dark grey, low = LR light grey), separately for contingent (CR) and non-contingent (NCR) trials. (**C)** Probability to choose the high reward task in CR trials (p(HR)), as a function of how many trials passed since the last reward contingency switch. Participants chose below chance (50%) on trials immediately following a contingency switch (‘perseveration’), and then quickly switched to choosing the HR task on subsequent trials. All error bars depict the SEM.

We first assessed whether error rates or reaction times (RT) differed between the two sets of SR mappings (labelled mapping X, Y), or the reward contingency conditions (CR, NCR). We expected no effects of SR mappings, but did expect CR trials to be faster and/or more accurate than NCR trials. The average error rate across all subjects was 5.89% (SEM = 0.74%). Thus, subjects were able to perform accurately. There was no evidence for an effect of reward condition on error rates (Bayes Factor (BF10) = 0.88). Error trials were removed from all further analyses. A repeated-measures ANOVA on RTs including the factors SR mapping and contingency revealed evidence for the absence of any RT differences between the two contingency conditions (BF10 = 0.01, Fig. 2A). The absence of reward contingency effects is likely due to the long delay phase, which gave participants much time to prepare task execution, and the relative lack of time pressure during the task execution. We further found evidence for the absence of RT differences between the specific SR-mappings implemented in each trial (Fig. 1, BF10 = 0.01), showing that both SR-mappings were equally difficult to perform. There was moderate evidence for the absence of an interaction between task and reward contingency (BF10 = 0.23).

As a control analysis, we then assessed whether subjects showed choice biases towards one of the two SR mappings, which might indicate stable preferences and in turn affect fMRI analyses (see below). We found no choice bias in CR trials, 50.35% (SEM = 1.49%), which did not differ from 50%, BF10 = 0.18, BF10_r=1.41_ = 0.09). The same was true for NCR trials, 51.40% (SEM = 1.75%), BF10 = 0.24, BF10_r=1.41_ = 0.12, indicating that subjects did not exhibit strong preferences for specific SR mappings.

We then assessed performance in the reversal-learning (CR) paradigm. For this purpose, we computed how often subjects chose the highly reward (HR) task, and this value should be above 50% if they succeeded in learning the changing reward contingencies. Subjects chose the HR task in 61.10% (SEM = 1.74%) of the CR trials, which was above 50% (BF10 > 150). As a control analysis, we computed the number of HR outcomes in NCR trials, which should be 50% if choices were uncorrelated with outcomes. This was indeed the case, HR outcomes = 49.47% (SEM = 0.84%), t-test vs 50% (BF10 = 0.21). Importantly, we found CR trials to lead to HR outcomes more often than NCR trials (56.4%, SEM = 1.15%, BF10 > 150), demonstrating that subjects succeeded in choosing tasks strategically in CR trials to maximize their reward outcomes. In order to assess how they maximized their outcomes, we computed the probability to switch to a different task just after receiving a high or a low reward (p_switchHR_, p_switchLR_). As expected, we found subjects to stay in the task that led to a high reward on the previous trial, p_switchHR_ = 15.71% (SEM = 2.60%), and switch away from the task that led to a low reward on the previous trial, p_switchLR_ = 71.08% (SEM = 3.20%, Fig. 2B). We found no such difference in NCR trials, p_switchHR_ = 53.12% (SEM = 2.83%), p_switchLR_ = 52.85% (SEM = 2.46%). An ANOVA with the factors reward contingency (CR, NCR) and reward outcome (HR, LR) identified a main effect of reward outcome on p_switch_ (BF10 > 150), no main effect of contingency (BF10 = 1.85), and a strong interaction (BF10 > 150). This demonstrates that subjects employed a win-stay loose-switch strategy and had clear reward-expectations selectively in CR trials, and that reward outcomes in NCR trials had no immediate effect on task choices.

We then assessed learning of changing reward contingencies in CR trials, by computing the probability to choose the highly rewarded task (p_HR_), as a function of trials passed since the last contingency change. We expected subjects to systematically choose the LR task immediately following such a change (‘perseveration’), but then quickly learn about the new contingency mapping. Our results confirmed these expectations (Fig. 2C). While subjects rarely picked the HR task immediately following a contingency switch (p_HR_ = 32.48%, SEM = 2.28%), this value increased dramatically already on the subsequent trial (p_HR_ = 65.65%, SEM = 2.88%, paired t-test, BF10 > 150). There was no evidence for changes in p_HR_ on the following four trials, all BF_10_s < 0.36, demonstrating that learning occurred mostly within the first trial following a contingency switch. Lastly, we hypothesized that subjects stayed in the same task longer in CR trials, as compared to NCR trials, and found this to be the case (Supplementary Analysis 1).

### Multivariate decoding of reward outcome values

#### Baseline analysis

**Figure 3:**
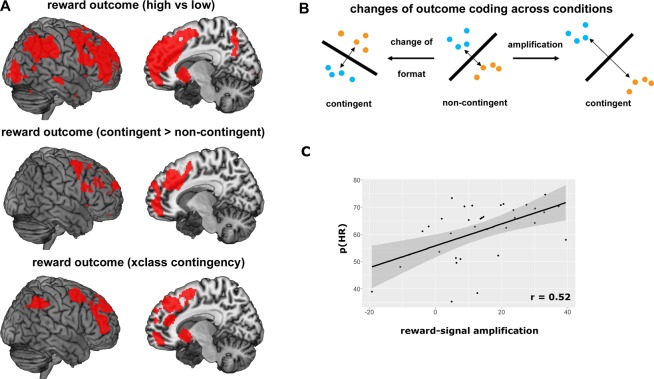
Reward-related brain activity. (**A)** Multivariate decoding of reward outcome value. Above: baseline decoding. Depicted are regions that encoded the value of reward outcomes (high vs. low, combined across CR and NCR trials). The regions identified were used as masks for the following analyses. Results are displayed at p < 0.05 (FWE corrected). Middle: regions showing stronger outcome coding in contingent (CR) than in non-contingent (NCR) trials. Below: regions encoding reward values using similar formats in both contingency conditions, as tested using a cross-classification (xclass) analysis. (**B)** Amplification vs change of format of neural coding. Most regions identified in A showed both stronger decoding in CR trials, and similar formats across both contingency conditions. This is compatible with an amplification or gain increase of neural codes. In the middle, a hypothetical example of a pattern decoding is depicted. High reward trials are depicted as blue, low reward trials as orange dots. The classifier fits a decision boundary to separate the two distributions. If this code changes between the two contingency conditions (left), decoding might still be possible at similar accuracy levels as before, but a classifier trained on NCR trials will be unsuccessful in classifying CR trials. If this code is amplified in the CR condition however (right), the same patterns become more easily separable. The same classifier will be will be successful in both conditions and accuracies will increase. See^27^ for more information. (**C)** Correlation of reward signal amplification and successful performance. This plot shows the correlation between the degree of reward signal amplification (accuracy in CR trials – accuracy in NCR trials), and successful performance in CR trials (probability to choose the high reward task, p(HR)). Each dot is one subject, and the line depicts a fitted linear function with 95% confidence intervals (gray area).

We first set out to determine whether outcome contingency affects the neural coding of outcome magnitude. For this purpose, we identified brain regions encoding outcome values (high vs low) at the time of feedback presentation (*baseline decoding*, collapsing across CR and NCR trials). We found an extensive network to encode outcome values including striatal subcortical brain regions, as well as large parts of the prefrontal and parietal cortex (Fig. 3A, mean accuracy = 60.52%, SEM = 1.11%). This contrast does not only capture specific reward value signals, it might also reflect effects *caused by* differences in reward outcomes, like attention or motor preparation. In order to address at least some of these confounding factors, we regressed RTs out of the data before performing MVPA^25^, but found no strong effects on our results (Supplementary Fig. 1).

#### Differences in outcome coding

Subsequently, we assessed whether these outcome signals were modulated by reward contingency, hypothesizing that contingent rewards showed stronger decoding results than non-contingent rewards. We repeated the same decoding analysis separately for CR and NCR trials and assessed whether any region from the baseline analysis showed stronger outcome coding in CR (mean accuracy = 64.17%, SEM = 2.01%) than in NCR trials (mean accuracy = 54.32%, SEM = 1.24%, using a within-subjects ANOVA and small-volume correction, p < 0.001 uncorrected, p < 0.05 FWE corrected). We found the striatum, bilateral lateral PFC, dACC, anterior medial PFC, and IPS to show stronger reward outcome coding for contingent rewards. The opposite contrast (NCR > CR) yielded no significant results (p < 0.001 uncorrected, p < 0.05 FWE corrected). This effect cannot be explained by differences in outcome value per se, as the reward magnitude did not differ across the conditions, only contingency did.

#### Similarities in outcome coding

In a last step, we assessed whether any of the brain regions identified in the baseline analysis showed similar coding across contingency conditions, using a cross-classification approach (see Materials and Methods for more details^26,27^). We trained a classifier on CR trials, and tested it on NCR trials, and vice versa, and found the striatum, lateral and medial PFC, dACC, and IPS to encode rewards similarly across conditions (mean accuracy = 55.52%, SEM = 1.10%). Cross-classification direction had no effect on these results (Supplementary Analysis 2). This pattern of results suggests that the neural code for different reward outcomes did not change across contingency conditions, yet outcome signals were still stronger in CR than in NCR trials. This points to an amplification or gain-increase of reward-related signals through contingency^27^. Interestingly, post-hoc analyses revealed that the difference in decoding accuracies between CR and NCR trials (i.e. the strength of the signal-amplification), correlated positively with successful performance in CR trials (Fig. 3C), r = 0.52, 95%CI = [0.26, 0.76] (for more details see Supplementary Analysis 3). The more subjects amplified their contingent reward-representations, the more they succeeded in performing the reversal-learning task, which links outcome coding to behavior.

### Multivariate decoding of tasks

#### Maintenance period

**Figure 4:**
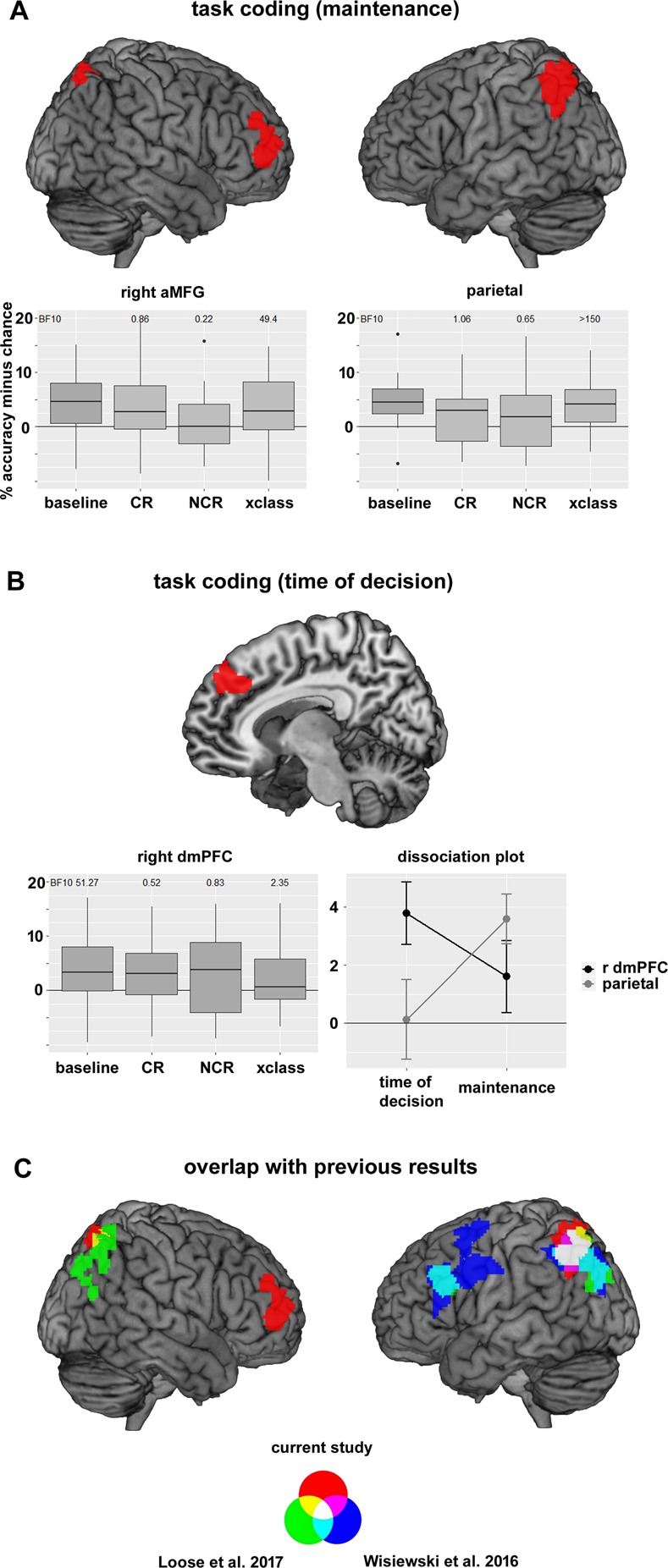
Task coding. (**A**) Task coding during maintenance. Results from the baseline decoding analysis are depicted above. Two clusters passed the significance threshold (p < 0.001 uncorrected at the voxel level, p < 0.05 FWE corrected at the cluster level), one in the parietal cortex, and one in the right anterior MFG. Accuracies were then extracted for the contingent (CR), non-contingent (NCR), and contingency cross-classification (xclass) task decoding analyses. Results can be seen in the boxplots. Above the plots, Bayes factors (BF10) of a t-test vs. chance level are shown. BF10 for the baseline analysis is not reported, as this analysis was used to define the ROIs, and running additional statistical tests on this data would constitute double dipping. (**B)** Task coding at the time of decision-making. Above, the dmPFC ROI used in this analysis (from^29^) is depicted. The box plot depicts results from our data in this ROI, for all four analyses performed (baseline, CR, NCR, xclass). The dissociation plot depicts a double dissociation between two ROIs (right dmPFC, as defined using data from^29^, and the left parietal cortex, as defined using data from^17^), and two time points in the trial (time of decision-making, maintenance). All error bars represent SEM. (**C)** Overlap with previous results. Results from the current study (red) are overlain on previous findings from^17^ (blue), and^16^ (green). All results are based on task decoding analyses (searchlight decoding, radius = 3 voxels, C = 1, chance level = 50%), albeit with different specific tasks being contrasted in each study. Despite this fact, all three studies find task information around the intraparietal sulcus. Findings in the PFC are less consistent.

*Baseline analysis:* For the task coding, we expected to see similar contingency effects as for the reward outcome signals. In a first analysis, we focused on the task maintenance period (from ‘choose’ cue onset to task screen onset, see Materials and Methods for more details, and^28^ for a similar approach). During this time, subjects maintain the abstract SR mapping they want to implement, without yet being able to implement specific motor actions. As in the outcome decoding analysis, we first combined CR and NCR trials to identify all regions encoding tasks (*baseline* decoding, see also^16^). We found two brain regions to maintain information about specific SR mappings, the left posterior parietal cortex (mean accuracy = 54.61%, SEM = 0.65%), spanning over the midline into the right parietal cortex, and the right anterior middle frontal gyrus (aMFG, mean accuracy = 54.66%, SEM = 0.89%, see Fig. 4A, Table 1). The parietal cluster identified here partly overlapped with the parietal cluster identified in the outcome decoding analysis, suggesting parietal involvement in both reward and task processing.

**Table 1:** Baseline task decoding.

MNI coordinates (peak)						
Brain region	Side	Cluster size	Mean accuracy (SEM)	X	Y	Z
parietal cortex	Bilateral	2427	54.61% (0.65%)	−10	−60	60
anterior MFG	Right	955	54.66% (0.89%)	32	58	18

Results are shown for a statistical threshold of p < 0.001 (uncorrected) at the voxel level and p < 0.05 (FWE corrected) at the cluster level. Chance level is 50%.

#### Differences in task coding

If contingent outcomes indeed enhance task coding, we should see higher accuracies in CR, than in NCR trials. To test this, we repeated the task decoding analysis separately for CR and NCR trials, and extracted accuracy values in the two ROIs found in the baseline task decoding analysis. We found no task information in the parietal cortex in these two analyses (CR: 51.29%, SEM = 0.91%, BF10 = 1.06; NCR: 51.73%, SEM = 1.44%, BF10 = 0.64), found no evidence for stronger task coding in CR than in NCR trials (BF10 = 0.16), and no evidence for stronger task coding in NCR than in CR trials (BF10 = 0.19). A similar pattern of results was found in the right aMFG (CR: 51.79%, SEM = 1.37%, BF10 = 0.85; NCR: 50.48%, SEM = 1.35%, BF10 = 0.22; CR > NCR: BF10 = 0.40; NCR > CR: BF10 = 0.10). Thus, we find no evidence for an effect of reward contingency on task representations, despite the fact that behavior clearly differed between the two contingency conditions, and that contingency has been found to modulate the coding of reward outcomes. As these two analyses are based on only half as many trials as the baseline analysis, a reduction in statistical power could explain the absence of any differences. In order to test this, we performed an additional control analysis, in which we showed that task coding is modulated by outcome magnitude (but not by outcome contingency), demonstrating that modulatory effects can be found in principle in this data-set with the same statistical power (Supplementary Analysis 4).

#### Similarities in task coding

As in the outcome decoding analysis, we also tested whether task representations were similar across the two contingency conditions, using a cross-classification approach. We found both the parietal cortex (54.03%, SEM = 0.76%, BF10 > 150), as well as the right aMFG (53.71%, SEM = 1.16%, BF10 = 49.39) to show similar task coding across contingency conditions. We also tested whether results from this cross-classification differed from the baseline accuracies, finding moderate evidence for an absence of any differences (parietal cortex BF10 = 0.23, aMFG BF10 = 0.25). These results show that the parietal cortex and aMFG encode tasks using a general format that is similar across reward contingency conditions.

#### Time of decision-making

Our experimental design allows us to independently assess task-related signals both at the time of decision-making and the subsequent maintenance period, as these were separated by a jittered delay (see Materials and Methods for more details). We thus repeated the same task decoding analyses at the time of decision-making. For this purpose, we used the right dmPFC cluster found previously^29^ as a ROI, which was found to encode task information at the time of decision-making. Despite the differences in experimental design, we found task information in the baseline analysis (53.76%, SEM = 1.07%, BF10 = 51.27, Fig. 4B) here as well. Accuracies were not above chance in CR trials (51.95%, SEM = 1.83%, BF10 = 0.52) and NCR trials (52.45%, SEM = 1.71%, BF10 = 0.83), and did not differ from each other (BF10 = 0.15). We found anecdotal evidence for successful task cross-classification in this region (52.03%, SEM = 0.98%, BF10 = 2.35), although the baseline and xclass analyses still did not differ (BF10 = 1.64). Interestingly, the dmPFC cluster partly overlapped with results from the reward outcome decoding.

Additionally, we found a double dissociation in task coding between the right dmPFC and left parietal cortex (Fig. 4B, both ROIs defined a priori from previous experiments^17,29^), with the former only encoding tasks at the time of decision-making, and the latter only encoding tasks during intention maintenance. An ANOVA using the factors ‘time in trial’ (time of decision vs intention maintenance) and ‘ROI’ (right dmPFC vs left parietal cortex) revealed moderate evidence for a time x ROI interaction (BF10 = 5.39). Furthermore, the right dmPFC only encoded chosen SR mappings at the time of decision (BF10 = 51.27), but not during intention maintenance (BF10 = 0.68). Conversely, the left parietal cortex only encoded chosen SR mappings during intention maintenance (BF10 > 150), but not at the time of decision (BF10 = 0.19). This suggests a temporal order of task processing in the brain, with task information first being encoded in dmPFC, and then in parietal cortex (but see^30^). Additionally, a post-hoc analysis revealed that decoding accuracies in the dmPFC at the time of decision-making correlated positively with trait impulsivity (Supplementary Analysis 3).

#### Exploratory analyses

In order to test the generalizability of our results, we repeated the same analyses on several a-priori ROIs, taken from two previous experiments^16,17^, which tested for effects of cognitive control, and free choice on task coding during a maintenance period, respectively. Overall, we were able to replicate the above effects in these a-priori defined ROIs, although findings were much more consistent in parietal than in prefrontal brain regions (Fig. 4C, Supplementary Analysis 5). In a similar vein, we also assessed task information in the multiple demand network^31,32^, and were able to replicate our findings in the parietal cortex (see Supplementary Analysis 6 for more information). An additional exploratory analysis also revealed task information in the orbitofrontal cortex (Supplementary Analysis 7), which is in line with recent findings on OFC function^33,34^. One might further reason that one’s choice history only mattered in CR trials, but not in NCR trials, and that participants should thus be more motivated to encode past choices in CR trials. We directly tested this notion in an additional exploratory analysis. The analysis revealed that parietal cortex, right aMFG, and right dmPFC all encoded past choices, although there was no evidence for stronger coding of past choices in CR trials in these regions (Supplementary Analysis 8).

#### Control analyses

It has been shown previously that RTs might affect task decoding results, leading to false-positive findings^25^. Although others failed to replicate this effect^35^, and we found no RT effects on the group level, we decided to conservatively control for RT effects on the single-subject level nonetheless,. After regressing RT-related effects out of the data for each subject, we still found the parietal cortex to encode specific SR mappings (54.61%, SEM = 0.65%, BF10 > 150), and also found task coding in the cross-classification analysis (54.03%, SEM = 0.76%, BF10 > 150). The same was true for the aMFG (54.66%, SEM = 0.89%, BF10 > 150; and 53.71%, SEM = 1.16%, BF10 = 23.38 respectively). Results in the baseline and xclass analysis were equal in both regions, BFs10 < = 0.30. These results thus mirror the main analysis above, showing that RT-related variance cannot explain task decoding results in our experiment.

In order to validate the decoding procedure, we also extracted task decoding accuracies from a region not involved in performing this task, the primary auditory cortex. As expected, we found accuracies not to differ from chance level in this region (49.64%, SEM = 0.93%, BF10 = 0.13), showing that the task decoding analysis was not biased towards positive accuracy values and specific to the regions reported.

Lastly, we performed two control analyses to directly assess possible biases in our decoding procedure (Supplementary Analyses 2 & 9), and a control analysis assessing the effects of error rates and/or choice biases on our task decoding results (Supplementary Analysis 10). None of these factors was found to affect our results.

## Discussion

Here, we investigated whether controlling reward outcomes modulates the neural coding of either outcomes or tasks. Subjects performed a probabilistic reward reversal-learning task, in which outcomes were contingent on specific choices. They also performed a free choice task with non-contingent reward outcomes, in which outcomes were not under their direct control. Although we found reward contingency to modulate outcome valuation, contrary to our expectations we found no effects on choice implementation. Furthermore, we found two main brain regions to be crucial for encoding tasks and reward outcomes: the right dmPFC and the left parietal cortex (around the IPS). The dmPFC was found to encode chosen tasks at the time of decision-making, and simultaneously encoded reward outcome values, emphasizing its role in both value-related with intentional control processes. While the parietal cortex encoded reward outcomes at the time of decision-making, it encoded chosen tasks during a subsequent maintenance phase. We found a double dissociation between both regions, with the dmPFC encoding tasks only at the time of decision-making, and the parietal cortex only during intention maintenance.

Much previous research on the effects of reward motivation on cognition investigated the effects of reward prospect^36^. These findings demonstrated that positive reinforcement often improves cognition, as compared to no reinforcement at all. However, an equally important and often overlooked property of reinforcement is the degree of control we have in reaching it. Sometimes, an action will cause outcomes in a fairly clear way (e.g. hitting a light switch), at other times, that link will be less close (e.g. refreshing your Facebook timeline). Previous work has shown that the strength of such action-outcome contingencies modulates the neural processing of reward outcomes^11^. Specifically, activation levels in subcortical reward-related brain regions differ when rewards are contingent on performing specific instructed actions^37^, or contingent on the choice whether to act^38^, as compared to non-contingent rewards. Here we extend these findings, by showing that the neural coding of reward outcomes changes depending on their contingency on specific choices between different tasks (for more information on how this differs from ‘whether’ choices see^39^).

Outcomes (and related processes) are encoded more strongly if they are contingent on task choices, as compared to when they are not. Their representational format further does not change strongly across contingency conditions. This is compatible with an amplification of outcome representations through contingency (Fig. 3B), where representations do not change but become more separable in neural state space (see^13^ for a similar argument). This is in line with predictions from gain-theories of motivation, which suggest that subcortical dopaminergic neurons can modulate their gain^6^, making them more or less sensitive to changes in rewards (see also^40^). Here, we demonstrate such gain increases in subcortical dopaminergic regions and beyond. This effect is unlikely to reflect simple motor processes, as regressing RTs out of the data did not alter results. It might be related to reward processing, but given the current data we cannot fully exclude that other related (e.g. attentional) processes contribute as well. Crucially, the strength of this gain increase was correlated with successful performance, the more subjects increased neural gain, the more successful they were in performing the reversal-learning task. Thus, our data demonstrates how reward-related signals changed, and that they are behaviorally relevant for successful performance.

Importantly, in order for this value signal to lead to actual rewards, chosen behavior has to be implemented as intended first^41^. One might thus expect contingency to lead to stronger task shielding and coding^12^, as the costs of confusing the two similar tasks are potentially high. However, we found no evidence for such effects. On the contrary, we found evidence for a similar coding of SR mappings across both contingency conditions. This finding informs current debates on the nature of task coding in the brain^27^. On the one hand, some have argued for flexible task coding especially in the fronto-parietal cortex^32,42^, often based on the multiple-demand network theory^31^. This account predicts that task coding should be stronger when task demands are high^32^, or when correct performance is rewarded^19^. Despite our efforts to replicate these findings in our data-set, we found no evidence for an influence of reward contingency on task coding. This was despite the fact that behavior differed between these conditions and that value-related signals were affected by reward contingency. One might argue that our analysis had insufficient statistical power to detect true effects, but a control analysis revealed that we can detect task coding differences in this data-set in principle, making this explanation unlikely.

On the other hand, some have argued that the same task representations could be used in multiple different situations (i.e. ‘multiplexing’ of task information), and that this allows us to flexibly react to novel and changing demands^14^. Multiplexing predicts that task information should be invariant across different contexts, which has been shown previously^16–18^. Here, we extend these findings, by showing that SR mappings are encoded in a format that is similar across contingency conditions in both frontal and parietal brain regions, strengthening the idea of multiplexing of task information in the brain. One possible alternative explanation for this finding might be that subjects were highly trained in performing the two tasks, and were at their performance ceiling. This might make a modulation of task coding too small to detect. Although we cannot fully exclude this interpretation, we want to point out that contingency did have robust effects on behavior. Also, most related previous experiments trained their subjects, those that found modulatory effects^19,32^ and those that did not^17^. We thus believe this alternative explanation to be unlikely. A second alternative explanation might be that outcome contingency does not affect the coding of maintained tasks, but still affects the coding of other task-relevant variables. For instance, it might be that a separate set of brain regions encodes beliefs about the current task state (“Am I performing the ‘good’ task now?”), and that this information is then affected by outcome contingency. This explanation is compatible with our results, although we demonstrate that the coding of specific SR mappings remains insulated from any such effects. Overall, our task decoding results are in line with the idea of multiplexing of task information in the brain. Future research will have to test more systematically which environmental conditions lead to multiplexing of task information in the brain, and which do not.

One key region identified here was the dmPFC. It is supports effort-based foraging choices^29^, and here we show its involvement in a reward reversal-learning task. The dmPFC is important for cognitive control, supporting rule and action selection^43^, and processing uncertainty^44^. It has further been associated with valuation processes, anticipating positive outcomes^45^, and encoding reward prediction errors^46^. In this experiment, we demonstrated that the dmPFC is specifically involved in encoding SR mappings only at the time at which a choice is made, other regions later maintain that choice outcome until it can be executed. We also demonstrated the dmPFC to encode outcome values at the same time. Please note that we do not claim this value signal to only represent the magnitude of reward outcomes, it might also represent related processes (e.g. attention). Nevertheless, the cause of this effect are different outcome values, and this highlights the importance of the dmPFC in linking valuation to strategic decision-making, suggesting how it might support goal-directed behavior^47^.

The second key region identified in this experiment was the left parietal cortex (IPS). The parietal cortex is a key region for cognitive control^48^, and might have a more general function in encoding relational information^49^. More specifically, the parietal cortex encodes conditional links between tasks and their outcomes (“If I perform mapping X now, I will get a high reward”^23^). Here, we demonstrate that the left IPS also encodes conditional links between stimuli and responses (SR-mappings, “If I see a guitar, I will press the blue button.”, see also^16^). There is an ongoing debate whether tasks are represented in a purely declarative format, or whether they also incorporate motor information for optimal performance^50^. Our results are compatible with the latter, as the IPS is shown to encode relations between stimuli and responses in our task. Given that our experiment was not optimized to differentiate these alternative forms of representation, more research will be needed on this issue.

The IPS is also a part of the multiple demand network^31^, a set of brain regions characterized by their high flexibility to adapt to changing demands. Previous work on non-human primates demonstrated that the prefrontal cortex flexibly switches between representing different control-related information within single trials^51^. Our results show that the parietal cortex in humans exhibits similar flexibility^23^, it encodes both control-related and value-related variables. This provides further evidence for its flexibility, and it will be interesting to assess how the parietal cortex links value-related and control-related variables in future experiments. Given its involvement in foraging behavior^22^, the previous choice and outcome history might affects current choice representations in this brain region. Future studies optimized to investigate this question will help shedding more light on this issue.

In sum, we assessed whether controlling outcomes affects outcome and task processing in the brain. By comparing choices that are informed by expected outcomes as well as choices that are not, we linked largely parallel research on ‘free choice’ and value-based decision-making. While we found strong effects on outcome processing, we found no such effects on choice implementation. Our results further highlight the importance of both the dmPFC and parietal cortex in bridging valuation and executive processes in the brain.

## Materials and Methods

### Participants

A total of 42 subjects participated in this experiment (20 males, 21 females, 1 diverse). The average age was 22.6 years (min = 18, max = 33 years), 41 subjects were right-handed, one was left-handed. All subjects had normal or corrected-to-normal vision and volunteered to participate. Subjects gave written informed consent and received between 45€ and 55€ for their participation. The experiment was approved by the ethics committee of the Ghent University Hospital and was performed in accordance with the Declaration of Helsinki. Seven subjects showed excessive head movement in the MR scanner (>4 mm) and were excluded.

### Experimental design

#### Trial structure

The experiment was programmed using PsychoPy (version 1.85.2, psychopy.org, RRID:SCR_006571^52^). Each trial started with the presentation of a fixation cross centrally on-screen (300 ms, Fig. 1A). This was followed by the presentation of a choice cue (‘CHOOSE’, 600 ms), which instructed subjects to freely choose one of two tasks to perform in this trial. After a variable delay period (2000–6000ms, mean duration = 4000 ms), the task screen was presented for a total of 3000 ms, irrespective of RT. Similar to^29^, the task screen consisted of a visual object presented centrally on screen (Fig. 1B). This object was picked pseudo-randomly out of a pool of 9 different objects in 3 categories: musical instruments, furniture, means of transportation. Below, 4 colored squares were presented (magenta, yellow, cyan, gray), with the square positions being mapped onto 4 buttons, operated using the left and right index and middle fingers. Subjects were given the option to choose which of two sets of stimulus-response (SR) mappings to apply to the presented object (e.g. mapping ‘X’ = means of transportation → magenta, furniture → yellow, and musical instruments → cyan. mapping ‘Y’ = means of transportation → cyan, furniture → magenta, and musical instruments → yellow, the grey button was never task-relevant and included merely to balance left and right button presses). Thus, one button was correct for each task in each trial, and subjects were instructed to react as quickly and accurately as possible. Choices were inferred from the pressed buttons. We use the term ‘task’ here to also refer to different links between stimuli and responses, and we do not claim that different cognitive processes are required to perform each of these mappings. Importantly, the position of the colored buttons on screen was pseudo-randomized in each trial, preventing mapping-specific motor attention effects^53^ and preparation of specific motor responses before the onset of the task screen. Furthermore, which set of S-R-mappings was called mapping X and mapping Y was counter-balanced across subjects. After the task screen offset, a reward feedback was presented (image of 1€ coin (high reward, HR), 10€cent coin (low reward, LR), or red circle (no reward), 400 ms). After a variable inter-trial-interval (4000–14000 ms, geometrically distributed, mean duration = 5860 ms), the next trial began.

#### Reward conditions

Subjects were rewarded for correct performance on every trial. There were a total of two different reward conditions: contingent rewards (CR) and non-contingent rewards (NCR). In the NCR condition, the chosen reward in each trial was determined randomly. Irrespective of the chosen task, subjects had a 50% chance of receiving a high and a 50% chance of receiving a low reward (Fig. 1C). Subjects were instructed to choose tasks randomly in this condition, by imagining flipping a coin in their head on each trial^18^. In the CR condition, subjects performed a probabilistic reward reversal-learning paradigm, similar to^24^. In each trial, one task led to a high reward with an 80% and a low reward with a 20% probability (HR task). These probabilities were reversed for the other task (LR task). Subjects were merely instructed that reward contingencies were stable across a few trials, but could change over time, and needed to infer the current contingency from the trial-by-trial reward feedback. After choosing the HR task for 3 consecutive trials, reward contingencies reversed without warning with a chance of 50% in each subsequent trial (similar to^24^). At the end of the experiment, 15 trials were chosen randomly (both from CR and NCR trials), and whichever reward was earned in these trials was paid out as a bonus payment to the subjects. This ensured that subjects were motivated in each trial and condition.

This reward manipulation was designed to vary the degree of control over choice outcomes. Choices in CR trials directly affected reward outcomes, which made expected outcomes a highly relevant task feature to take into account during decision-making. Choices in NCR trials were unrelated to reward outcomes, and expected outcomes were irrelevant for decision-making. We ensured that the reward condition was uncorrelated to all other design variables (target stimulus, delay duration, button mapping, ITI duration), and previous trial conditions were not predictive of current trial conditions. This ensured that all trials were IID, and estimated neural signals were not confounded.

### Procedure and design

Subjects first performed a training session which lasted about 1 h10 min 1–5 days before the MR session. They learned to perform the task and completed 3 runs of the full experiment. This minimized learning effects during the MR session, which can be detrimental to cross-validated MVPA. In the MR session, subjects performed 5 identical runs of this experiment, with 60 trials each. Each run contained 2 blocks with CR and 2 blocks with NCR trials. The length of each block was between 10 and 14 trials, and all trials were separated by a long and variable ITI. CR and NCR blocks alternated and block order was counterbalanced across runs for each subject. Each block started with either ‘Contingent block now starting’ or ‘Non-contingent block now starting’ presented on screen (5000 ms). This mixed blocked and event-related design minimized cross-talk and interference between the reward conditions. Reward contingencies did not carry over from a one to the next CR block, in order to make each block independent from previous performance. Each run also contained 20% (n = 12) catch trials. In these trials, subjects were externally cued which SR mapping to implement, and the delay between cue and task execution was only 1000 ms. Catch trials were included to prevent subjects from choosing all tasks in a block at its beginning. For instance, in an NCR block, subjects could theoretically decide upon a whole sequence of choices at the beginning of that block (e.g. X, X, X, Y, X, Y, Y, X,…), and then only implement that fixed sequence in each trial. In order to encourage subjects to make a conscious choice in each individual trial, catch trials were included to frequently disrupt any planned sequence of task choices, making such a strategy less feasible. To maximize the salience of catch trials, correct performance always led to a high reward. Catch trials were excluded from all analyses.

#### Additional measures

After completing the MR session, subjects filled in multiple questionnaires. They answered custom questions (e.g., How believable were the instructions? Was one task more difficult than the other?) and the following questionnaires: behavioral inhibition/activation scale (BISBAS^54^), need for cognition (NFC^55^), sensitivity to reward/punishment (SPSRQS^56^), and impulsivity (BIS11^57^). We also acquired pupil dilation data while subjects performed the experiment in the MR scanner. Pupil dilation data is not the focus of the current paper, and is not reported.

#### Image acquisition

fMRI data was collected using a 3T Magnetom Trio MRI scanner system (Siemens Medical Systems, Erlangen, Germany), with a standard thirty-two-channel radio-frequency head coil. A 3D high-resolution anatomical image of the whole brain was acquired for co-registration and normalization of the functional images, using a T1-weighted MPRAGE sequence (TR = 2250 ms, TE = 4.18 ms, TI = 900 ms, acquisition matrix = 256 × 256, FOV = 256 mm, flip angle = 9°, voxel size = 1 × 1 × 1 mm). Furthermore, a field map was acquired for each participant, in order to correct for magnetic field inhomogeneities (TR = 400 ms, TE_1_ = 5.19 ms, TE_2_ = 7.65 ms, image matrix = 64 × 64, FOV = 192 mm, flip angle = 60°, slice thickness = 3 mm, voxel size = 3 × 3 × 3 mm, distance factor = 20%, 33 slices). Whole brain functional images were collected using a T2*-weighted EPI sequence (TR = 2000 ms, TE = 30 ms, image matrix = 64 × 64, FOV = 192 mm, flip angle = 78°, slice thickness = 3 mm, voxel size = 3 × 3 × 3 mm, distance factor = 20%, 33 slices). Slices were orientated along the AC-PC line for each subject.

### Statistical analysis

#### Data analysis: behavior

All behavioral analyses were performed in R (RStudio version 1.1.383, RRID:SCR_000432, www.rstudio.com). We first characterized subjects’ performance by computing error rates and reaction times (RT). We tested for potential effects of reward contingency on error rates using a Bayesian two-sided paired t-tests (using *ttestBF* from the BayesFactor package in R). Error trials (trials with wrong button presses, or with RTs < 300 ms) were then removed from the data analysis. In order to identify potential effects of the different SR mappings and reward contingency on RTs, we performed a Bayesian repeated measures ANOVA (using *anovaBF* from the BayesFactor package in R). This ANOVA included the factors SR mapping and reward contingency, and outputs Bayes Factors (BF) for all main effects and interaction terms. We did not expect to find RT differences between SR mappings, but did expect RTs to be lower in the CR condition, as compared to the NCR condition. All Bayesian tests were performed using the default prior (Cauchy prior, r = 0.707). We performed additional robustness checks using different priors (r = 1, r = 1.41), which did not change our results in most cases. When priors did affect the interpretation of results, these results are reported (e.g. BF10_r=1.41_), otherwise we only report results using the default prior.

The Bayesian hypothesis testing employed here allows quantifying the evidence in favor of the alternative hypothesis (BF10) *and* the null hypothesis, allowing us to conclude whether we find evidence for or against a hypothesized effect, or whether the current evidence remains inconclusive^58^. We considered BFs between 1 and 0.3 as anecdotal evidence, BFs between 0.3 and 0.1 as moderate evidence, and BFs smaller than 0.1 as strong evidence against a hypothesis. BFs between 1 and 3 were considered as anecdotal evidence, BFs between 3 and 10 as moderate evidence, and BFs larger than 10 as strong evidence for a hypothesis.

Given that subjects were free to choose between the two tasks, some subjects might have shown biases to choosing one of the two SR mappings more often (although that would not have led to a higher overall reward, if anything biases should lower overall rewards). In order to quantify biases, we computed the proportion of trials in which subjects chose each SR mapping, separately for the CR and NCR conditions, and tested whether this value differed from 50% using a two-sided Bayesian t-test. The output BF was interpreted in the same way as in the previous analysis.

Choices in CR trials were assessed by quantifying how well subjects performed the probabilistic reversal-learning paradigm. If subjects were able to reliably determine which of the two tasks was currently the HR task, they should have chosen that task more often than expected by chance (50%). Thus the proportion of HR task choices (p_HR_) in CR trials is our main measure of how successful subjects were in performing the task. This measure was compared to chance level using a one-sided Bayesian t-test. We also computed this measure as a function of trials that passed since the last contingency switch. We expected subjects to systematically choose the LR task immediately following such a change (‘perseveration’), but then quickly learn about the new contingency mapping. We tested whether p_HR_ changed across trials by using Bayesian paired t-tests. Furthermore, we assessed the earned reward outcomes, expecting p_HR_ to be higher in CR than in NCR trials (where it should be 50%). This was tested using a paired one-sided Bayesian t-test.

In order to describe the strategies employed to maximize reward outcomes, we computed the probability to switch to a different task immediately following a high or a low reward (p_switchHR_, p_switchLR_). We expected subjects to follow a “win stay loose switch” strategy (WSLS), staying in a highly rewarded task and switching away from a lowly rewarded task. To test this, we performed a two-factorial Bayesian ANOVA, including the factors reward outcome (HR, LR) and reward contingency (CR, NCR). We expected to see a main effect of outcome on p_switch_, and an interaction, as WSLS should be specifically applied to CR trials only.

#### Data analysis: fMRI

fMRI data analysis was performed using Matlab (version R2014b 8.4.0, RRID:SCR_001622, The MathWorks) and SPM12 (RRID:SCR_007037, www.fil.ion.ucl.ac.uk/spm/software/spm12/). Raw data was imported according to BIDS standards (RRID:SCR_016124, http://bids.neuroimaging.io/), and was then unwarped, realigned and slice time corrected.

#### Multivariate decoding of reward outcomes

In a first step, we assessed whether we can replicate previous findings demonstrating contingency effects on reward processing^10,11^. For this purpose, we estimated a GLM^59^ for each subject. For each of the 5 runs we added regressors for each combination of reward value (HR vs LR) and contingency (CR vs NCR). All regressors were locked to the reward feedback onset, the duration was set to 0. Regressors were convolved with a canonical haemodynamic response function (as implemented in SPM12). Estimated movement parameters were added as regressors of non-interest to this and all other GLMs reported here.

Baseline decoding: In a next step, we performed a decoding analysis on the parameter estimates of the GLM. A linear support-vector classifier (SVC^60,61^), as implemented in *The Decoding Toolbox*^62^, was used with a fixed regularization parameter (C = 1). We performed searchlight decoding^20,28^, which looks for information in local spatial patterns in the brain and makes no a prior assumptions about informative brain regions. Searchlight radius was set to 3 voxels, and we employed run-wise cross-validation. We contrasted HR trials (from both CR and NCR trials) with LR trials (again from CR and NCR trials). The resulting accuracy maps were normalized to a standard space (Montreal Neurological Institute template as implemented in SPM12), and smoothed (Gaussian kernel, FWHM = 6 mm) in order to account for potential differences in information localization across subjects. Group analyses were performed on the accuracy maps using voxel-by-voxel t-tests against chance level (50%). A statistical threshold of p < 0.0001 (uncorrected) at the voxel level, and p < 0.05 (family-wise error corrected) at the cluster level was applied, which is sufficient to rule out inflated false-positive rates^63^. Any regions surpassing this threshold were used as masks for the following decoding analyses (an approach used previously^16^). The baseline reward decoding is likely partly driven by underlying univariate signal differences, and we do not claim that results reflect differences in response patterns only. This approach does allow us to compare results directly to task-related analyses, which employed the same analysis strategy. The main aim of this analysis was to identify all regions involved in processing reward outcomes. We are not primarily interested in which regions will be found per se, but rather focus on whether reward-related signals will be modified by contingency.

Differences in reward outcome coding: Although the baseline decoding analysis should have the maximum power to detect any outcome-related brain regions, results do not allow us to conclude whether outcome processing differed between CR and NCR trials. For this purpose, we performed an additional two searchlight decoding analyses. In the first, we again contrasted high and low reward trials, now only using data from CR trials. In the second, we used only data from NCR trials. If contingent rewards indeed enhance encoding of reward outcomes in the brain, we should see higher accuracies in the CR than in the NCR decoding analysis. Please note, that we only used half the number of trials as before, thus considerably reducing the signal-to-noise ratio in these analyses. We thus expected lower statistical power and smaller effects. Also, whereas the baseline decoding results themselves might be driven by e.g. differences in attentional processing between high and low rewards, looking at *differences* between CR and NCR trials much reduces the impact of any such unspecific differences on decoding results, which are driven more by differences between contingency. We still cannot fully exclude that unspecific processes contribute to these results, however. Lastly, focusing on *differences* between conditions avoids potential issues with double dipping.

Similarities in in reward outcome coding: Previous work demonstrated that only some brain regions show a contingency-related modulation of value signals^10^, and we thus tested whether any brain regions encoded reward outcomes similarly across the contingency conditions. In an additional searchlight decoding analyses, we trained a classifier to discriminate between high and low reward outcomes in the CR condition, and tested its performance in the NCR condition, and vice versa. This resulted in two accuracy maps per subject, which were averaged and then entered into a group analysis just like in the previous analyses. Importantly, only brain regions where the same hyperplane can be used to differentiate neural patterns across both contingency conditions (i.e. in which patterns do not differ substantially) will show above-chance accuracies in this analysis. This so-called cross classification (xclass) analysis provides positive evidence to identify regions that encode reward outcomes independent of the contingency manipulation used here (see also^26^).

#### Multivariate decoding of tasks

We then employed the same analysis strategy described above to investigate possible effects of outcome contingency on task coding as well. Two GLMs were estimated for each subject, one modelling task-related brain activity at the time of decision-making, and one modelling activity during a subsequent maintenance phase. It has been shown that formation and maintenance of intentions rely on partly dissociable brain networks^64^, and our design allowed us to estimate independent signals related to both epochs as they were separated by a variable inter-trial-interval.

In the first GLM (GLM_maintenance_), for each of the 5 runs we added regressors for each combination of chosen task (mapping X, mapping Y) and reward contingency (CR, NCR). All 4 regressors were locked to the cue onset, the duration was set to cover the whole delay period, during which subjects maintained their task representations. Due to the jittered delay period duration, the modelled signals were dissociated from the task execution and feedback presentation (see also^17^). These boxcar regressors were then convolved with a canonical haemodynamic response function.

A second GLM was estimated (GLM_decisiontime_), in order to extract task-specific brain activity at the time subjects made their choice which of the two tasks to perform. This GLM only differed in the time to which regressors were locked. Although the cue suggested that subjects should make a task choice at that point in time, there is no strong way of controlling the exact point in time at which choices were made in any free-choice paradigm, and they might have been made earlier in principle. It has been shown before that under free choice conditions, subjects choose a task as soon as all necessary information to make a choice is available^24,29^. In this experiment, this time point is the feedback presentation of the previous trial, and regressors in this analysis were locked to that event. At this point, subjects can judge whether they e.g. chose the HR or LR task and determine which of the two tasks to perform in the next trial. We used this approach successfully in a previous experiment^29^, and all further task decoding analyses were performed on both GLMs.

Baseline decoding: The task decoding analyses followed the same logic as the reward outcome analyses described above. We first performed a searchlight decoding analysis (radius = 3 voxels, C = 1), contrasting parameter estimates for mappings X and Y in all trials (CR and NCR combined). This analysis has the maximum power to detect any brain regions containing task information, which can be notoriously difficult^65^. Resulting accuracy maps were normalized, smoothed (6 mm FWHM), and entered into a group analysis (t-test vs chance level, 50%). Results were thresholded at p < 0.001 (uncorrected) at the voxel level, and p < 0.05 (family-wise error corrected) at the cluster level. Again, regions surpassing this threshold were used to define functional regions-of-interest for the following decoding analyses^16^.

Differences in task coding: In order to assess whether task coding is modulated by reward contingency, we repeated the decoding analysis separately for CR and NCR trials. If contingent rewards indeed increase task shielding in the brain, we should see higher accuracies in the CR than in the NCR decoding analysis. This effect should be especially pronounced if both tasks are similar and easily confused, which is the case in our experiment. Again, the power of these analyses is considerably lower than for the baseline analysis.

Similarities in task coding: Some previous work suggests that tasks are encoded in a context-independent format in the brain^17,18^. Here, we again used cross-classification (xclass) by training a classifier on CR trials and then testing it on NCR trials (and vice versa). Any brain regions showing above chance decoding accuracies in this analysis provides positive evidence of task coding that is similar across contingent vs non-contingent reward conditions. This procedure also ensures that task-related signals are not confounded by potential differences in e.g. cognitive load or expected reward across the CR and NCR conditions, as classifiers are trained and tested only *within* one contingency condition.

#### Exploratory analyses

We also performed a number of exploratory analyses, testing whether our results would generalize to other regions of interested (Supplementary Analyses 5–7), and whether we found any evidence for representation of past choices (Supplementary Analysis 8). We also assessed potential correlations between our behavioral measures, questionnaire scores, and fMRI results (Supplementary Analysis 3).

#### Control analyses

In order to further corroborate the validity of our results, we performed a number of control analyses. It has been pointed out before that RT effects might partly explain task decoding results^25^, although others were unable to show any such effects^29,35^. Irrespective of the group-level results of testing for RT differences between contingency conditions or tasks, we decided to conservatively control for RTs effects at the single-subjects level as well. First, we repeated the GLM estimation, only adding reaction times as an additional regressor of non-interest. We then repeated the main decoding analyses, and tested whether accuracy values differed significantly. If RTs indeed explain our task decoding results, we should see a reduction in decoding accuracies when RT effects were regressed out of the data.

Then, we performed a ROI decoding analysis on a brain region that is not related to task-performance in any way, expecting accuracies to be at chance level. We chose the primary auditory cortex for this purpose, defined using the WFU_pickatlas tool (https://www.nitrc.org/frs/?group_id=46, RRID: SCR_007378). If our effects are specific to cognitive-control related brain regions, we should see chance level results here.

We also assessed whether our decoding procedure was biased towards positive accuracies (Supplementary Analysis 9), tested whether error rates or potential choice biases affected task decoding results (Supplementary Analysis 10), and tested whether cross-classification direction had an influence on results (Supplementary Analysis 2).

## Supplementary information

Supplementary Material

## Data Availability

The raw data generated and analyzed in this study can be found online on the Open Science Framework, https://osf.io/9j7ra.
