# Supplementary material for: Outcome contingency selectively affects the neural coding of outcomes but not of tasks
Source: Sci Rep. 2019 Dec 18;9:19395. doi: 10.1038/s41598-019-55887-0 (PMC6920387; doi:10.1038/s41598-019-55887-0)
Supplement: Supplementary file 1 — Supplementary Material [file 41598_2019_55887_MOESM1_ESM.pdf]

Outcome contingency selectively affects the neural coding of outcomes but not of tasks

David Wisniewski[1], Birte Forstmann[2], Marcel Brass[1]

1 Department of Experimental Psychology, Ghent University, Belgium

2 Integrative Model-Based Cognitive Neuroscience Research Unit, University of Amsterdam, The Netherlands

Supplementary Material

Supplementary Analyses

Supplementary Analysis 1

In CR trials, subjects were incentivized to repeat the same SR mapping in consecutive trials, while this was not the case for NCR trials. In order to assess this, we computed the distribution of run lengths for each subject, i.e., the number of trials subjects chose to consecutively perform the same task, separately for CR and NCR trials. If task choices were not based on the random reward outcomes, this distribution can be expected to follow an exponential distribution [1]. Conversely, the run length for CR trials should be longer than in NCR trials, as participants are expected to repeat a highly rewarding task more often. Differences in run lengths were tested using a one-sided Bayesian t-test.

The average run length in CR trials was 2.54 trials (SEM = 0.08), which was indeed longer than in NCR trials (1.95 trials, SEM = 0.07, BF10 >150, see also Figure Supplementary Analysis 1). Interestingly, we found no evidence for repetition biases in NCR trials, which have been reported before [1]. Run length in NCR trials was equal to what would be expected if subjects chose tasks randomly (BF10 = 0.20, $BF10_{r=1.41}$ = 0.10), which is necessary, but not sufficient to demonstrate truly random task choices.

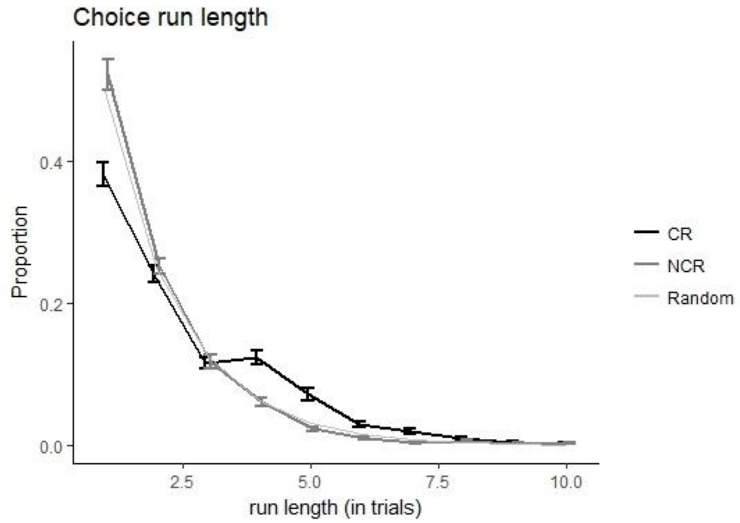

Figure Supplementary Analysis 1. Run length distributions. Depicted are the distributions of run lengths for CR (black) and NCR (dark grey) trials. The expected distribution if choices were random, or at least not guided by reward outcomes, is depicted in light grey.

Supplementary Analysis 2

In order to test whether the training set had an influence on the results of our cross-classification analyses, we performed an additional control analysis. For the reward outcome decoding analysis (high vs low outcomes), we first extracted accuracy maps separately for both cross-classification directions (CR->NCR, NCR->CR), and used a whole-brain paired t-test to assess whether any brain regions show stronger decoding results for a specific direction. Such differences might arise in the case if e.g. reward outcomes were distributed differently in CR and in NCR trials, which might lead to the classifier being trained on a biased data set. We deliberately used a lenient threshold for this analysis ($p < 0.01$, FWE corrected at the cluster level, $p < 0.05$), which has been shown to inflate false-positive rates [2]. Despite this fact, we found no significant results. The same procedure was then applied to the task decoding analysis, in which we also performed a cross classification across contingency conditions. Again, no significant results were found. Cross-classification direction thus had no detectable effect on our decoding results.

Supplementary Analysis 3

An additional exploratory analysis was performed to correlate performance, questionnaire measures, and decoding accuracies. Several key correlations were assessed using Bayesian correlation

analysis (using *bayes.cor.test* form the BayesianFirstAid package) in order to estimate whether they deviated from zero. We report the estimated correlation (r), the probability of the correlation being above or below zero ($p_{r>0}$, $p_{r<0}$), and the 95% credible intervals (95% CI). If the interval does not contain zero, the correlation is larger or smaller than zero.

Correlating performance with decoding results: First, we assessed whether performance in the tasks was correlated with decoding results. We only found successful performance in CR trials to be correlated with the degree of reward signal amplification in CR trials (as compared to NCR trials), r = 0.52, $p_{r>0}$ > 0.99, 95% CI = [0.26, 0.76]. The more reward signals were amplified, the more successful subjects were in performing the reversal learning task, which demonstrates the behavioral relevance of the reward signal amplification.

Correlating performance with questionnaire results: We found success in CR trials to be negatively correlated with motor impulsivity, r = -0.37, pr<0 = 0.98, 95% CI = [-0.66,-0.08]. The relation to impulsivity was specific to motor impulsivity, we found no strong relation of CR performance with either attentional or non-planning impulsivity. This finding suggests that (motor) impulsive subjects were worse in performing the reversal-learning task (see also [3]). We found no correlation of success with either sensitivity to reward (r = 0.11, $p_{r>0}$ = 0.74, 95% CI = [-0.23, 0.44]), or the need for cognition (r =0.20, $p_{r>0}$ = 0.87, 95% CI = [-0.12, 0.52]), despite the fact the need for cognition has previously been associated with reward decision-making [4].

Correlating decoding with questionnaire results: We found task decoding accuracies in the dmPFC at the time of decision-making to be positively correlated with non-planning impulsivity, r = 0.33, $p_{r>0}$ = 0.92, 95% CI = [0.018, 0.63], suggesting that impulsive subjects had stronger task representations right after all necessary information was presented to make a choice. Non-planning impulsivity has been linked to reward decision-making on the behavioral level previously [3], but this result suggests that a similar relation might be present at the neural level as well. Overall, the relation of impulsivity and

performance / decoding results was unexpected, and future research should be targeted more at explaining how impulsivity affect the neural basis of reward decision-making.

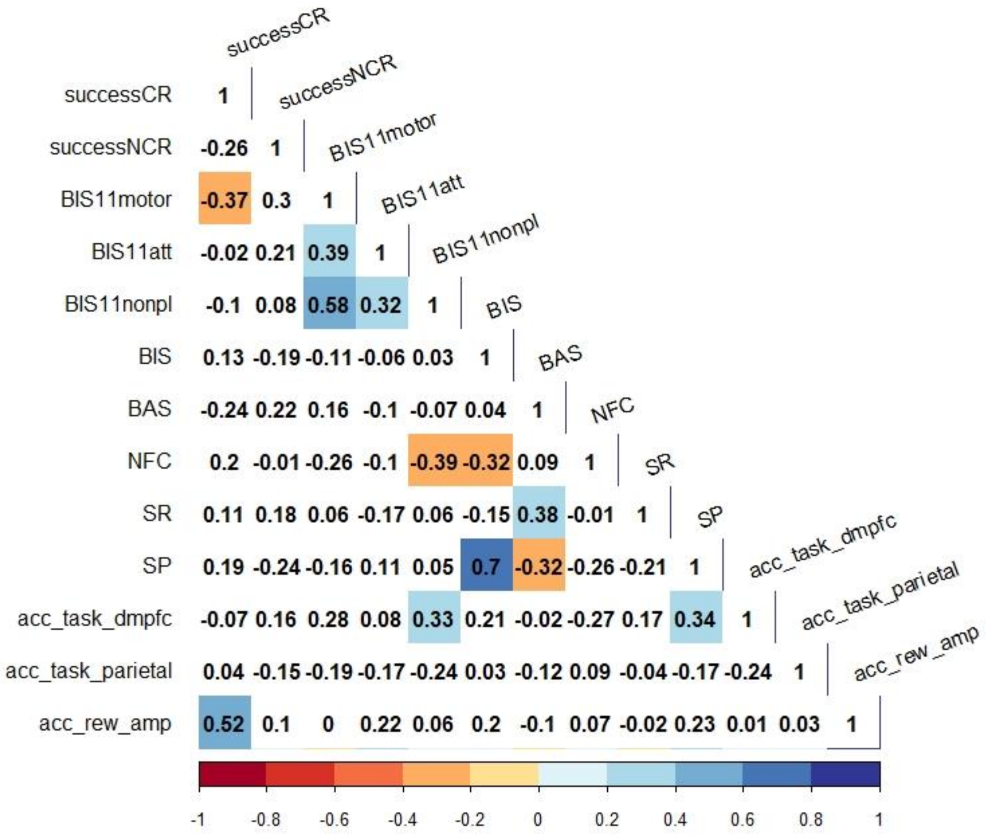

Figure Supplementary Analysis 2: Correlation analysis. Depicted are pairwise correlations (estimated using *bayes.cor.test* form the BayesianFirstAid package) of: % high reward choices in CR trials (successCR), % high reward outcomes in NCR trials (successNCR), motor impulsivity (BIS11motor), attentional impulsivity (BIS11att), non-planning impulsivity (BIS11nonpl), behavioral inhibition (BIS), behavioral approach (BAS), need for cognition (NFC), sensitivity to reward (SR), sensitivity to punishment (SP), decoding accuracies in the baseline task decoding analysis in the dorso-medial PFC (acc_task_dmpfc) and parietal cortex (acc_task_parietal), and an index of reward signal amplification (acc_rew_amp). This index was computed by subtracting accuracy values from the reward outcome decoding in NCR trials, from the accuracy values in CR trials. Only regions that showed successful cross-classification of reward signals across contingency conditions were included here. This procedure leads to a global measure of how strongly reward signals were amplified in the CR condition, as compared to the NCR condition. The plot was generated using the *corrplot* package in R. Number show the correlation coefficients. Correlations in white cells did not differ from zero (i.e. the 95% CI included zero), correlations in colored cells did.

Supplementary Analysis 4

Given that decoding tasks separately in CR and NCR trials has considerably less power than decoding tasks in both CR and NCR trials together, we performed an additional control analysis to determine if the reduced power explains the absence of any differences between task coding in CR and NCR trials.

Previous research demonstrated that task signals in the brain are modulated by associated reward outcome magnitude, i.e. tasks that lead to a high reward show stronger coding than tasks that lead to no reward [5]. Based on this, we expected to find a similar effect in our experiment as well. We tested whether tasks directly following a HR outcome were encoded more strongly than tasks directly following a LR outcome. For this purpose, we estimated another set of GLMs for each subject, only now splitting trials into following HR vs LR (instead of CR vs NCR trials). In all other respects, this analysis was identical to the task decoding presented in the main body of the paper. We then extracted decoding accuracies from the parietal, right aMFG, and dmPFC clusters used in the main analysis, separately for HR task decoding and LR task decoding. Using Bayesian t-tests vs chance and a paired Bayesian t-test, we assessed whether there were any differences between these accuracies. We expected task coding to be stronger in HR trials especially at the time of decision making, as this time point is closer to the reward feedback presentation than the maintenance period.

At the time of decision making, we found strong task coding in the dmPFC in HR trials (60.67%, $BF10 > 150$), but no task coding in LR trials (51.07%, $BF10 = 0.33$). Crucially, there was strong evidence for a difference between these values ($BF10 = 31.89$). During the maintenance period, the parietal cortex showed task coding in the HR condition (54.95%, $BF10 = 5.83$), but not in the LR condition (52.32%, $BF10 =0.59$). We found no evidence for any difference between these values however ($BF10 = 0.66$). In the right aMFG a similar picture emerged. We found task coding in HR trials (56.07%, $BF10 = 14.36$), but not in LR trials (52.82%, $BF10 =1.51$), and no difference between these values ($BF10 = 1.03$).

Thus, we find evidence for differences in task coding, although the evidence is stronger at the time of decision-making than the maintenance period. This demonstrates that our analysis approach can in principle detect differences in task coding in the current data-set, yet we still fail to find any such differences between contingent and non-contingent trials.

Supplementary Analysis 5

We assessed task information in a number of a priori defined ROIs. First, we attempted to replicate results from some previous experiments [6,7]. Both of these experiments assessed whether task coding is modulated by external variables. While Wisniewski et al. (2016) looked into effects of free choice on task coding, Loose et al. (2017) looked into effects of cognitive control demands on task coding. Both studies found a fronto-parietal network to encode task sets and found them to be independent of the experimental manipulation employed. Given that the tasks employed in these studies were different from the one used here (e.g. addition vs subtraction of two numbers), a replication of these findings in the current dataset would show a strong generalizability of our results. We thus extracted functional ROIs from both papers (Wisniewski et al. 2016: left parietal cortex, left PFC, Brodman area 8; Loose et al. 2017: left parietal cortex, left PFC), and extracted accuracy values for all voxels within the ROI for all four decoding analyses performed (baseline, CR, NCR, xclass), which were then averaged. One-sided Bayesian t-tests across subjects were performed to assess whether they were above chance.

We were able to replicate Loose and colleagues' left parietal results (baseline BF10 = 133.69; CR BF10 = 0.68; NCR BF10 = 0.54; xclass BF10 = 33.17). Although somewhat weaker, we also replicated their right parietal results (baseline BF10 = 8.49; CR BF10 = 0.77; NCR BF10 = 0.14; xclass BF10 = 8.10). However, we were unable to detect task information in left PFC (baseline BF10 = 0.49; CR BF10 = 0.21; NCR BF10 = 0.44; xclass BF10 = 0.29), which is in line with the original paper, where PFC findings were also somewhat less robust. Additionally, we were able to replicate Wisniewski and colleagues' left

parietal finding (baseline BF10 > 150; CR BF10 = 0.80; NCR BF10 = 0.47; xclass BF10 = 87.28), as well as left BA8 (baseline BF10 = 9.3; CR BF10 = 0.39; NCR BF10 = 0.36; xclass BF10 = 3.09), but not the left PFC (baseline BF10 = 0.59; CR BF10 = 0.37; NCR BF10 = 0.16; xclass BF10 = 0.38). Thus, we find task coding to be contingency-independent even in ROIs that were defined using independent datasets. We further show that task information is most consistently found in the parietal cortex, but less so in prefrontal cortex.

Supplementary Analysis 6

Some previous work suggested that task information can be found in the multiple demand (MD) network [8], and that task coding in this network changes flexibly with changing task demands [9]. In order to test whether this was also the case in our dataset, we extracted accuracy values for all four decoding analyses (from bilateral functional MD ROIs (provided by [10]), specifically the anterior insula (aINS), cerebellum, inferior frontal gyrus pars opercularis (IFGop), intraparietal sulcus (IPS), middle frontal gyrus (MFG), pre-central gyrus (precG), supplementary and pre-supplementary motor area (SMA/preSMA), as well as thalamus. We then tested whether decoding accuracies were higher in CR than in NCR trials, and/or whether task coding was contingency-independent in these regions. Averaging across all MD regions, we found strong evidence for the presence of task information (52.23%, SEM = 0.61%, BF10 = 69.08, see Figure below). We found no evidence for a higher accuracy in CR, as compared to NCR trials (BF10 = 0.37). Furthermore, we found task coding to be contingency-independent (52.02%, SEM = 0.67%, BF10 = 14.52). Accuracies in the baseline and cross-classification analysis did not differ (BF10 = 0.19). Looking at individual MD regions, we found task information in the aINS (52.25%, SEM = 1.00%, BF10 = 3.23), IPS (52.83%, SEM = 0.72%, BF10 = 131.02), MFG (52.44%, SEM = 0.90%, BF10 = 8.26), precentral gyrus (52.48%, SEM = 0.87, BF10 = 9.86), but not in the cerebellum (50.85%, SEM = 0.90%, BF10 = 0.44), IFGop (52.11%, SEM = 1.02%, BF10 = 2.31), SMA/preSMA (51.48%, SEM = 1.07%, BF10 = 0.77), and thalamus (50.58%, SEM = 1.06%, BF10 = 0.29). None of these regions showed a higher

accuracy in CR than in NCR trials (BFs10 <= 0.60). However, in all of those regions the accuracy in the baseline and xclass analyses was equal (BFs10 <= 0.28). This suggests that the MD network encodes tasks in a contingency-independent fashion, and shows that the current context does not affect task coding in the MD network. Also, not all parts of the MD network seemed to be encoding tasks in our experiment.

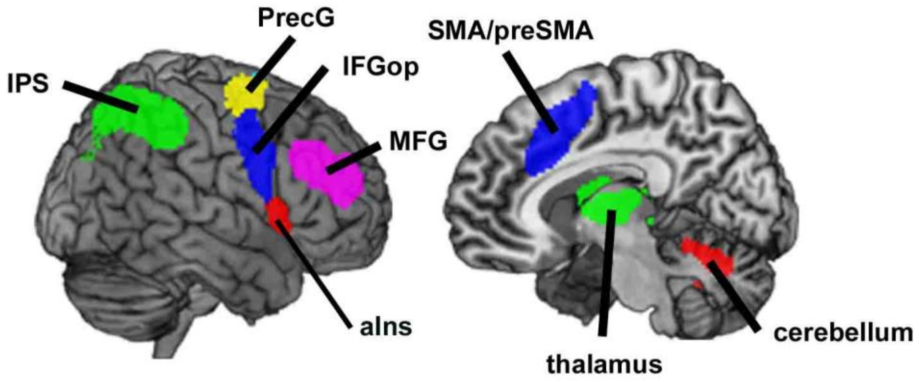

Figure Supplementary Analysis 5. Task information in the multiple demand (MD) network. Functional ROIs are provided by Fedorenko, Duncan, & Kanwisher (2013), specifically the anterior insula (aINS), cerebellum, inferior frontal gyrus pars opercularis (IFGop), intraparietal sulcus (IPS), middle frontal gyrus (MFG), pre-central gyrus (precG), supplementary and pre-supplementary motor area (SMA/preSMA), as well as thalamus.

Supplementary Analysis 7

Recent findings demonstrated the orbitofrontal cortex (OFC) to encode the location of participants in an abstract cognitive task space [11,12]. Based on these findings, one might hypothesize the OFC to also encode task information in this experiment. To test this notion, we performed an additional exploratory analysis. We first defined an OFC region of interested, using the same approach as [13]. We then assessed whether the OFC encodes tasks at the time of decision-making, and during maintenance (in the baseline decoding analysis). We did not find task information at the time of decision-making (51.22%, SEM = 0.90%, BF10 = 0.84), but did find the OFC to encode tasks during the maintenance

period (52.44%, SEM = 0.44%, BF10 = 26.43). We then explored the maintenance period further, and found evidence for successful cross-classification across contingency conditions (51.63%, SEM = 0.75%, BF10 = 3.05). Additionally, we were able to decode tasks in CR trials (52.56%, SEM = 1.02%, BF10 = 5.51), but not in NCR trials (50.42%, SEM = 1.11%, BF10 = 0.29), although we found no evidence for any differences between these two conditions (BF10 = 1.14). Overall, the OFC results are similar to our main findings in the parietal cortex, showing that the OFC indeed tracks task information.

Supplementary Analysis 8

One major difference between CR and NCR trials is the role past choices play during decision-making. Our behavioral results show that choice history was only taken into account in CR, but not in NCR trials. Based on this, one might hypothesize that past choices should be encoded more strongly in CR than in NCR trials. To test this hypothesis, we performed an additional exploratory analysis. We first estimated a separate GLM for each participant, using regressors for each combination of task choices on trial t-1 and the reward condition: mapping X_{t-1_CR}, mappingX_{t-1_NCR}, mappingY_{t-1_CR}, mappingY_{t-1_NCR}. Regressors were time-locked to the choice cue onset, and duration was set to the whole delay phase, identical to our main analysis of the maintenance phase. In fact, the only difference between this and the main analysis was that we investigated previously performed, instead of currently maintained tasks. We then performed the same four decoding analyses on this data as before (baseline, CR, NCR, xclass), and would expect stronger coding in CR, as compared to NCR trials. Please note that the same analysis cannot be performed on the time of decision-making, as this time point is not separated from task execution by a variable jitter, and results might be confounded by attentional or motor processes, making them difficult to interpret.

We then assessed results in the same ROIs used in the main analysis (parietal cortex, right aMFG, right dmPFC). We found the parietal cortex to encode the past choice in the baseline analysis (53.18%, SEM = 1.12%, BF10 = 6.65). The same was true for the right aMFG (52.41%, SEM = 0.98%, BF10

= 4.89) and right dmPFC (53.02%, SEM = 0.98%, BF10 = 16.67). None of these regions showed task coding in CR trials only, NCR trials only, or the cross-classification analysis (all BF10s < 1.81). Crucially, none of these regions showed stronger coding in CR, as compared to NCR trials, all BF10s < 0.41). Overall, parietal cortex, right aMFG, and right dmPFC encode past choices, but results remain inconclusive with regards to differences between CR and NCR trials. There was no evidence suggesting these signals were modulated by outcome contingency. Future research optimized to investigate the coding of choice histories might shed more light on the coding format of past choices in these brain regions.

Supplementary Analysis 9

In order to assess whether our decoding procedure was biased towards positive accuracy values, we empirically estimated the chance level and tested if it was indeed 50% as we assumed. For this purpose, we performed a permutation analysis (n = 1000 permutations per subject, as implemented in the Decoding Toolbox, using the same regressors and contrasts as the baseline task decoding analysis) in order to estimate the null distribution of our data. We took the mean of the null distribution as our empirical estimate of the chance level, and tested whether it deviated from 50% (using a two-sided Bayesian t-test). If there were some global biases in our decoding procedure, chance level should deviate from 50%. The estimated chance level was 49.98%, which did not differ from 50% (BF01 > 150). Thus, comparing our decoding accuracies against a chance level of 50% was valid.

Supplementary Analysis 10

Although overall error rates were very low, and we found no evidence for persistent choice biases across our sample, there might still be individual subjects that do show e.g. high error rates, which might affect our task decoding results by decreasing signal-to-noise ratio. Although the effect of few outlier subjects should be small given our large sample size, we still chose to conservatively control

for such (unlikely) effects. We first excluded subjects with the highest error rates (more than 1.5*IQR above average, i.e. error rate > 13.92%), and then excluded subjects with the strongest choice biases (more than 1.5*IQR above average, i.e. percent task X choices > 61.99% or < 38.73%). We then tested whether each regressor in the task decoding analysis in all remaining subjects could be estimated from at least 6 trials. If a regressor could only be estimated from fewer trials, that run was excluded from the analysis due to the low signal-to-noise-ratio. Subjects in which more than 1 run was thusly excluded were altogether excluded from the analysis. These criteria were highly similar to the criterion used in [14], which proved an effective control. After excluding these subjects, we repeated the main four analyses (baseline, CR, NCR, xclass) on the remaining subjects and tested whether they differed from the analysis including all subjects.

Using these highly conservative exclusion criteria, we removed 2 subjects due to their error rate, 5 subjects due to their choice biases, and 4 subjects due to the small number of trials, leading to a sample size of 24 subjects. Even though statistical power was considerably lower because of the smaller sample size, we were still able to detect task information in the same ROIs as in the main analysis: parietal cortex baseline decoding (55.09%, SEM = 0.78%, BF10 >150), cross-classification (53.63%, SEM = 0.98%, BF10 = 60.25), and the same was true for the aMFG (54.74%, SEM = 1.09%, BF10 >150, and 53.53%, SEM = 1.35%, BF10 = 6.57, respectively). These results are even numerically larger than in the original analysis, and neither error rates not choice biases were found to affect the reported task decoding results.

Supplementary Figures

Supplementary Figure 1

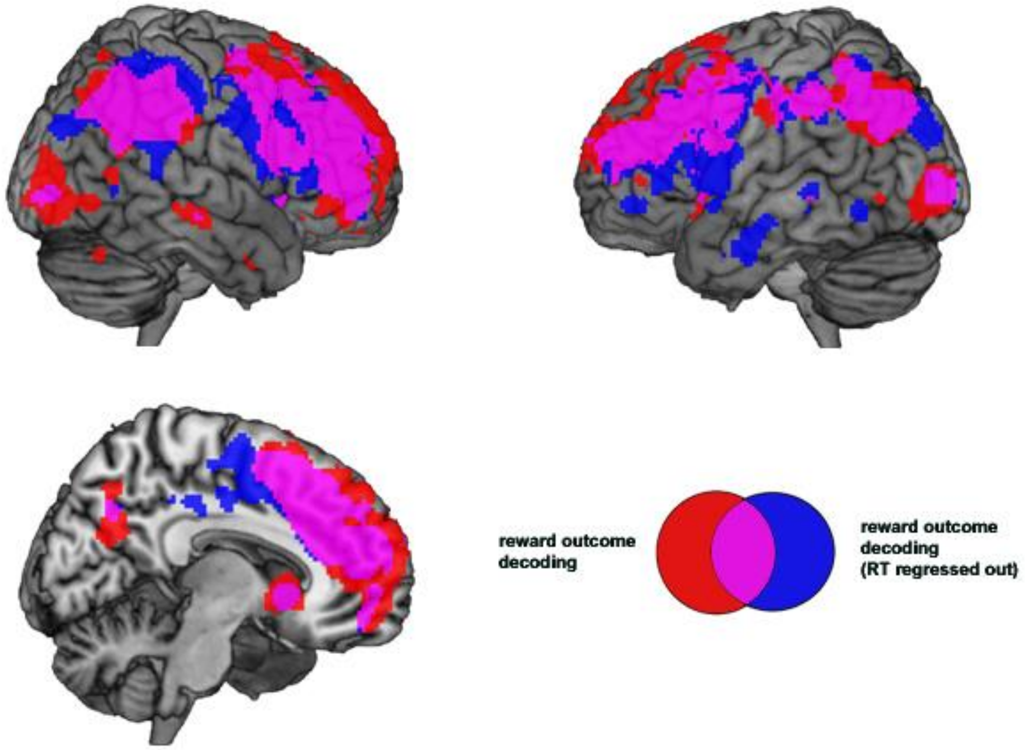

Supplementary Figure 1. Controlling RT-effects in reward outcome decoding. We repeated the reward outcome decoding analysis, using a similar first-level GLM to estimate signals (4 regressors: high contingent reward, low contingent reward, high non-contingent reward, low non-contingent reward, all locked to feedback onset). Additionally, we now added parametric regressors of non-interest capturing RT-related variance in the data. The rest of the analysis was identical to the reward outcome decoding analysis presented in the main body of the text. Results from the reward outcome decoding analysis (red), and the same analysis with RT-related effects regressed out of the data (blue) are depicted. As can be seen, the overlap (magenta) between both analyses is substantial. Results depicted at $p < 0.05$ (FWE, corrected at the voxel level). This indicates that controlling for RT did not strongly alter our results.

References

1. Arrington, C. M. & Logan, G. D. The cost of a voluntary task switch. *Psychological Science* **15**, 610–615 (2004).
2. Eklund, A., Nichols, T. E. & Knutsson, H. Cluster failure: Why fMRI inferences for spatial extent have inflated false-positive rates. *PNAS* **113**, 7900–7905 (2016).
3. de Wit, H., Flory, J. D., Acheson, A., McCloskey, M. & Manuck, S. B. IQ and nonplanning impulsivity are independently associated with delay discounting in middle-aged adults. *Personality and Individual Differences* **42**, 111–121 (2007).
4. Sandra, D. A. & Otto, A. R. Cognitive capacity limitations and Need for Cognition differentially predict reward-induced cognitive effort expenditure. *Cognition* **172**, 101–106 (2018).
5. Etzel, J. A., Cole, M. W., Zacks, J. M., Kay, K. N. & Braver, T. S. Reward Motivation Enhances Task Coding in Frontoparietal Cortex. *Cereb. Cortex* **26**, 1647–1659 (2016).
6. Wisniewski, D., Goschke, T. & Haynes, J.-D. Similar coding of freely chosen and externally cued intentions in a fronto-parietal network. *NeuroImage* **134**, 450–458 (2016).
7. Loose, L. S., Wisniewski, D., Rusconi, M., Goschke, T. & Haynes, J.-D. Switch-Independent Task Representations in Frontal and Parietal Cortex. *J. Neurosci.* **37**, 8033–8042 (2017).
8. Duncan, J. The multiple-demand (MD) system of the primate brain: mental programs for intelligent behaviour. *Trends in Cognitive Sciences* **14**, 172–179 (2010).
9. Woolgar, A., Hampshire, A., Thompson, R. & Duncan, J. Adaptive Coding of Task-Relevant Information in Human Frontoparietal Cortex. *Journal of Neuroscience* **31**, 14592–14599 (2011).
10. Fedorenko, E., Duncan, J. & Kanwisher, N. Broad domain generality in focal regions of frontal and parietal cortex. *Proceedings of the National Academy of Sciences* **110**, 16616–16621 (2013).
11. Wilson, R. C., Takahashi, Y. K., Schoenbaum, G. & Niv, Y. Orbitofrontal Cortex as a Cognitive Map of Task Space. *Neuron* **81**, 267–279 (2014).
12. Schuck, N. W., Cai, M. B., Wilson, R. C. & Niv, Y. Human Orbitofrontal Cortex Represents a Cognitive Map of State Space. *Neuron* **91**, 1402–1412 (2016).
13. Schuck, N. W. & Niv, Y. Sequential replay of nonspatial task states in the human hippocampus. *Science* **364**, eaaw5181 (2019).
14. Wisniewski, D., Reverberi, C., Tusche, A. & Haynes, J.-D. The Neural Representation of Voluntary Task-Set Selection in Dynamic Environments. *Cereb. Cortex* **25**, 4715–4726 (2015).
